# Noisy Cell-Size-Correlated Expression of Cyclin B Drives Probabilistic Cell-Size Homeostasis in Fission Yeast

**DOI:** 10.1016/j.cub.2019.03.011

**Published:** 2019-04-22

**Authors:** James O. Patterson, Paul Rees, Paul Nurse

**Affiliations:** 1 Cell Cycle Laboratory, The Francis Crick Institute, 1 Midland Road, London NW1 1ST, UK; 2 College of Engineering, Swansea University, Bay Campus, Fabian Way, Swansea SA1 8EN, UK; 3 Imaging Platform, Broad Institute of Harvard and MIT, 415 Main Street, Cambridge, MA 02142, USA; 4 Laboratory of Yeast Genetics and Cell Biology, Rockefeller University, 1230 York Ave, New York, NY 10065, USA

**Keywords:** cell size, cyclin-dependent kinase, CDK, cell division, cell growth, systems biology, cyclin, single-cell biology, cell division, mitosis

## Abstract

How cells correct deviations from a mean cell size at mitosis remains uncertain. Classical cell-size homeostasis models are the sizer, timer, and adder [1]. Sizers postulate that cells divide at some threshold size; timers, that cells grow for a set time; and adders, that cells add a constant volume before division. Here, we show that a size-based probabilistic model of cell-size control at the G2/M transition (P(Div)) can generate realistic cell-size homeostasis *in silico.* In fission yeast cells, Cyclin B^Cdc13^ scales with size, and we propose that this increases the likelihood of mitotic entry, while molecular noise in its expression adds a probabilistic component to the model. Varying Cdc13 expression levels exogenously using a newly developed tetracycline inducible promoter shows that both the level and variability of its expression influence cell size at division. Our results demonstrate that as cells grow larger, their probability of dividing increases, and this is sufficient to generate cell-size homeostasis. Size-correlated Cdc13 expression forms part of the molecular circuitry of this system.

## Highlights

• A size-correlated division probability can generate cell-size homeostasis
• Cyclin B concentration scales noisily with size in fission yeast
• Cells with stochastically suprathreshold cyclin B are the ones that divide
• A new tetracycline inducible promoter with linear dose response is developed

## Graphical Abstract

**Figure:**
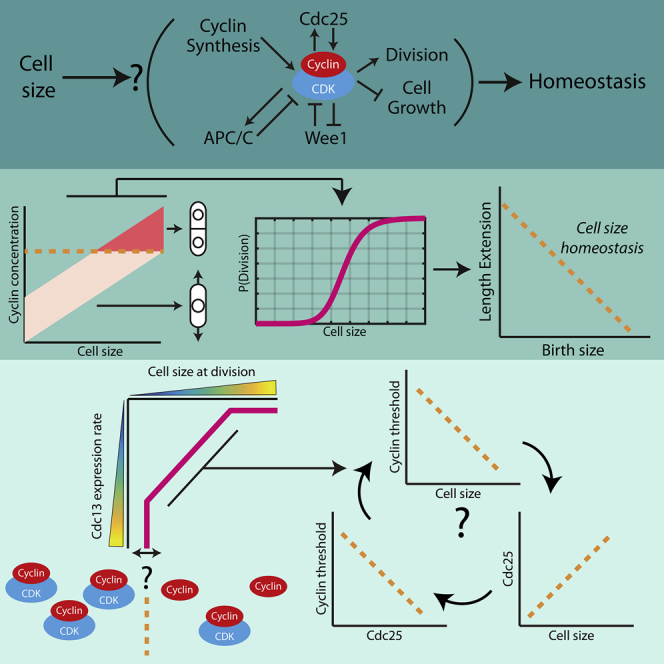

## Teaser

Patterson et al. show that a probabilistic model of cell-size control can generate cell-size homeostasis in fission yeast. Cyclin B levels scale noisily with cell size, providing a molecular correlate for the phenomenological model. Using a new inducible promoter, they show that cyclin B and cell-size variability scale proportionally.

## Results and Discussion

**Figure 1:**
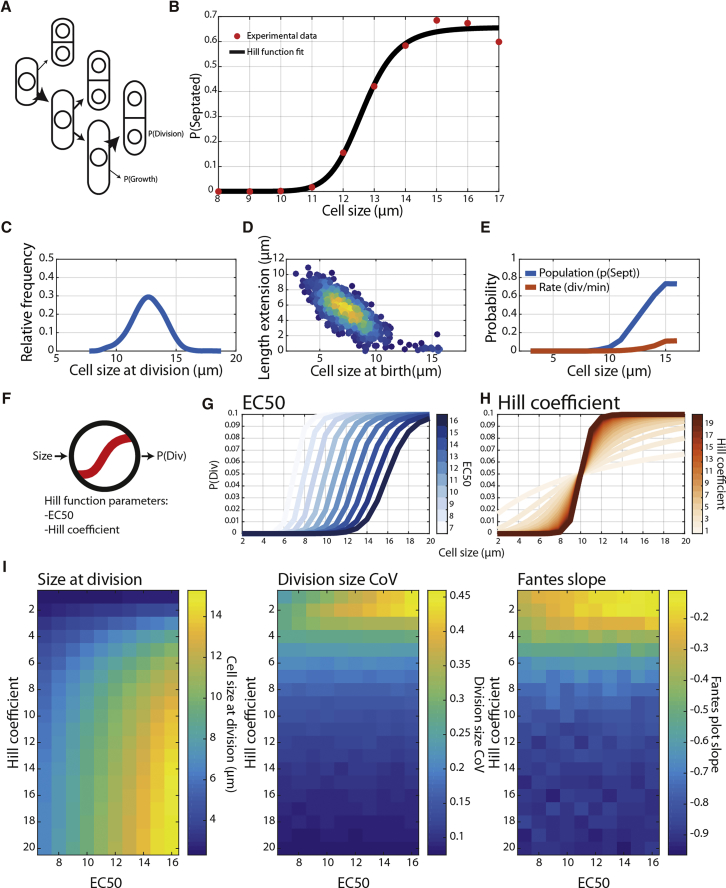
A P(Div) Model of Cell Size Control Generates Cell-Size Homeostasis (A) Schematic of the P(Div) model. The basis of the model is that as cells grow larger, their probability of division increases. (B) Plot of the fraction of septated cells (a surrogate for P(Div)) for WT cells grown in Edinburgh minimal media (EMM) at 32°C. Data were acquired on an Imagestream system following calcofluor staining. Red points indicate the proportion of cells within a 1 μm size bin that are septated. The black line represents a Hill curve fit to the red data points by non-linear fit within MATLAB. Hill coefficient = 10.25, EC_50_ = 12.6, N = 275087. (C) Relative frequency plot of cell size at division from simulated data. Simulations are initiated with 20 cells roughly at the mean birth size and run for 1,000 min. All cells grow according to an exponential function that results in size doubling within ∼120 min. Simulations result in >1,000 individual full cell cycles. The probability of cell division at a certain cell size is sampled from a Hill curve with a maximum probability of 0.1, EC_50_ of 14, and Hill coefficient of 14. (D) Fantes plot of cell-size homeostasis. Data points are colored by the density of points. The cell population is simulated as in (C). (E) P(Div) plots derived from simulation data. Div/min curve is not experimentally accessible, and P(Sept) curve is equivalent to data shown in (B). The cell population is simulated as in (C). (F) Generalized schematic of the P(Div) model as a dose response function with size as input and P(Div) as output. (G) Plot of a Hill function with Hill coefficient = 14 and EC_50_ varied. (H) Plot of a Hill function with EC_50_ = 10 and Hill coefficient varied. (I) Heatmaps of relevant extracted cell-size control parameters when Hill coefficient and EC_50_ are varied *in silico*. *In silico* cell growth proceeds as in (E). See also Figure S1, Table S1, and Data S1.

The fission yeast *Schizosaccharomyces pombe* is a good model for the study of cell-size control, with extensive genetic resources, a well conserved cell-cycle architecture, and an ability to efficiently correct cell-size deviations [2]. Previous molecular models of size control in *S. pombe* have focused on the size-dependent regulation of cyclin-dependent kinase (CDK) activity through tyrosine phosphorylation at the G2/M transition. These include molecular ruler type sizer models driven by the kinases Pom1 [3, 4] and Cdr2 [5] and the size-dependent accumulation of the CDK activator Cdc25 [6, 7]. However, a strain that cannot be regulated by these pathways due to an absence of a tyrosine phosphorylatable CDK [8] still maintains cell-size homeostasis [2]. This could be due to further regulation at the G2/M transition or possibly due to exposure of a cryptic G1/S size control [9]. A model proposed for budding yeast *S. cerevisiae* G1/S size control is based on the size-dependent dilution of the CDK inhibitor Whi5 [10]. However, a recent study that quantified cell-size homeostasis revealed that loss of Whi5 does not appear to affect cell-size fidelity and that classical regulators of the G2/M transition also play a role in correcting cell-size deviations [11]. In this paper, we consider the number of cells that are *not* dividing at some threshold size and have used a “probability of division” or P(Div) model of size control (Figure 1A). This model postulates that as cells grow larger, their probability of dividing increases. This type of model has been previously used to model the size at the division distribution of *S. cerevisiae* in an exponential growing population [12], and a similar model has also been proposed for bacterial size control [13, 14].

Fission yeast cells stop elongating during mitosis and septate at the same length at which they divide; thus, a metric for P(Div) can be calculated from the proportion of cells of a particular length that exist in septated and non-septated states. Cell length does not influence the length of time spent in a septated condition [15], and thus this metric will be equally proportional to the probability of dividing across all cell sizes. To measure P(Div) experimentally, an Imagestream analyzer was used to image cells, allowing cell size and septation state to be determined for ∼100,000 cells. By binning the acquired cells by size, the probability of division per size bin can be calculated (Figure 1B), and the P(Div) in wild-type (WT) cells thus calculated can be fitted to a Hill curve. These results indicate that a probabilistic dose-response mechanism can generate a realistic cell-size model. The P(Div) model is a specific version of the sizer model, which allows for a maximum rate of division (that is defined by the plateau of the Hill curve) to be set.

To investigate the P(Div) size model further, *in silico* simulations were carried out. A population of growing cells was triggered to divide by either an adder, deterministic sizer, timer, or the P(Div) model of size control (schematics of each shown in Figure S1A). An example of the *in silico* growing population is shown in Figure S1B. A comparison of the P(Div) model with deterministic sizer, adder, and timer models is shown in Figure S1C. The P(Div) model simulation generates a cell-size distribution with a realistic CoV of ∼9% (Figure 1C) [2]. The Fantes plot (a plot of birth length versus length extension) is a powerful tool to assess cell-size homeostasis [16]. The P(Div) size model generates a Fantes plot with a gradient of −0.85, demonstrating cell-size homeostasis (Figure 1D). This agrees with data from time-lapse measurements of cells [2]. This model allows some cells to divide without growing, albeit in small numbers, and so fully modeling experimental results also requires an additional minimal timer element [1].

The P(Div) plot shown in Figure 1B does not feature a time-dependent element. To extract the temporal P(Div) rate, we calculated it *in silico* (Figure 1E; STAR Methods). The degree of separation between the experimental P(Div) curve and the P(Div)/time curve is related to the time a cell spends septated. If septation and division were instantaneous, the curves would be equivalent. Thus, normalizing the non-temporal P(Div) size curve by the septation time allows the calculation of a P(Div) rate. The maximum probability of dividing per minute that generates the experimentally determined P(Div) curve can be calculated to be ∼0.15. These data demonstrate that the P(Div) model as applied here should be considered in addition to the sizer model for generating cell-size homeostasis in fission yeast.

The Hill function, described by three parameters (EC_50_, the point at which probability of division reaches 50% of the maximum; the Hill coefficient, a measure of curve ultrasensitivity; and maximum P(Div)), can model the experimentally derived P(Div) function (Figure 1B). The size distribution of *S. pombe* cells is altered in response to changes in nutrient conditions [17, 18]—the population mean size changes, but cell-size homeostasis is maintained. To investigate whether our model can accommodate a change in division size, single cell cycles were simulated using the P(Div) function for size control (Figure 1F) with varied EC_50_ values (Figure 1G) and Hill coefficients (Figure 1H). Varying both the EC_50_ and Hill coefficients can alter the mean cell size at division (Figure 1I—left heatmap). These simulations show that when a Hill coefficient is maintained >7, altering EC_50_ can easily shift cell size at division to different values. Importantly, both the CoV of size at division (Figure 1I—middle heatmap) and the strength of size homeostasis (Figure 1I—right heatmap) remain compatible with cell-size control when EC_50_ is altered. Such changes in EC_50_ without changes in Hill coefficient have been reported in molecular studies of kinase networks and could be mediated by changes in associated regulatory pathway activity [19].

**Figure 2:**
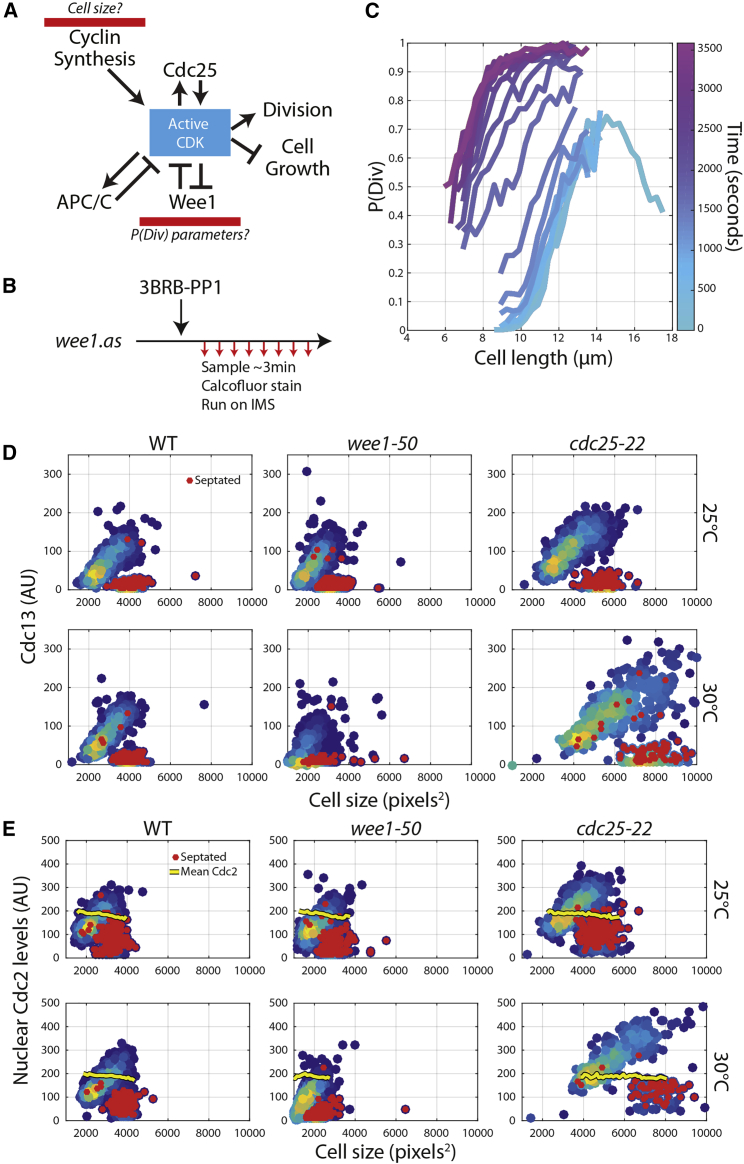
The Cell-Cycle Control Network Could Provide a Molecular Explanation for the P(Div) Model of Cell-Size Control (A) Potential molecular inputs that would explain the P(Div) model. (B) Schematic of the experiment. Wee1.as cells were grown in EMM at 25°C. Time 0 corresponds to the addition of 3BRB-PP1 to the culture flask. Cells were subsequently sampled and prepared for IMS acquisition every ∼3 min. (C) P(Div) curves from each sample time point with time of sampling from addition of 3BRB-PP1 indicated by the color of the line. N > 5,000 cells per line. (D) Cdc13 levels presented here are derived from mean nuclear intensity. Septation state is assigned from calcofluor stained images and is marked by red data points. Colors of other data points indicate the density of the data point (dark blue, few; yellow, many). Cells were grown in EMM and at permissive (25°C) and semi-permissive (30°C) as annotated. N > 500 cells for all strains. (E) Scatterplots of nuclear Cdc2-sfGFP levels versus cell size in annotated mutants and temperatures. The yellow line represents the rescaled mean intensity of Cdc2-sfGFP (window size, 100 pixels^2^). The mean has been rescaled so that it can be observed on the same plot as the scatter of mean nuclear Cdc2-sfGFP levels. Septation state is assigned from calcofluor stained images and is marked by red data points. Colors of other data points indicate the density of the data point. Cells were grown in EMM and at permissive (25°C) and semi-permissive (30°C) as annotated. N > 400 cells for all strains. See also Figure S2, Table S1, and Data S1.

The Hill function models switch like dose-response functions in biochemical systems, and it has been applied to the dose response of cyclin versus CDK activity [20, 21, 22, 23, 24]. As the output of the P(Div) size model is cell division, and given that Wee1 and Cdc25 have been shown to regulate cell division ultrasensitively, we hypothesized that alteration of Wee1 enzyme activity may alter the shape of the P(Div) function (Figure 2A). Implicit in this hypothesis is the idea that cyclin levels (specifically concentration) may be related to cell size. We made use of an engineered bulky ATP-analog-sensitive Wee1 allele [25], which allowed rapid alteration of Wee1 kinase activity. P(Div) size was determined after chemical Wee1 inhibition (Figure 2B). Cells progressed through mitosis and triggered septation and then after 20–30 min transitioned to a new P(Div) curve, with cells triggering septation at smaller cell sizes, effectively shifting the P(Div) curve’s EC_50_ lower (Figure 2C). This curve reaches a maximum P(Div) of 1.0 as it is out of steady state, and all larger cells are progressing through septation. This experiment demonstrates that Wee1 inhibition alters the parameters of the P(Div) function.

Wee1 and Cdc25 regulate the dose response of cyclin B levels versus CDK activity *in vitro* [21, 22, 24] and thus are likely to alter the dose response kinetics *in vivo* as well. If cyclin B (Cdc13) levels scaled with cell size across a range of sizes, it would provide a potential molecular input for the P(Div) function (Figure 2A). To assess this, we engineered strains containing Cdc13-sfGFP [26] in *wee1-50* and *cdc25-22* temperature-sensitive mutants. Cdc13-sfGFP levels were measured at permissive and semi-permissive mutant temperatures (Figure 2D). Size and cyclin levels correlate across a range of cell-size mutants and temperatures. Additionally, the maximum cyclin level appears to plateau at different cell sizes in the mutant strains, indicating a potential change in a cyclin threshold due to alteration of Wee1 or Cdc25 activity (Figure 2D and Figure S2).

While cyclin levels oscillate through the cell cycle, the *S. pombe* CDK1 (Cdc2) levels stay constant [27], with the Cdc2 protein becoming enriched in the nucleus of late G2 cells [28]. To investigate whether nuclear Cdc2 levels scale with cell size, we tagged Cdc2 with sfGFP in the background of both *wee1-50* and *cdc25-22* mutant cells and imaged as in Figure 2D. We confirmed that Cdc2-sfGFP mean levels are constant across the cell cycle (Figure 2E). In binucleates at the beginning of the cell cycle, nuclear Cdc2 levels start near 0. Cdc2 subsequently transitions into the nucleus in septating binucleates when Cdc13 is produced (Figure 2D). This is expected, as Decottignies, Zarzov, and Nurse [28] showed that cyclins are required for the accumulation of Cdc2 in the nucleus. As cells elongate, nuclear Cdc2-sfGFP scales with cell size. This is likely due to the accumulation of Cdc13, allowing more Cdc2 to become localized in the nucleus (Figure 2E). Thus, we propose that the nuclear accumulation of Cyclin/CDK complexes is size dependent and could serve as a molecular correlate of cell size in the P(Div) model. This proposal is consistent with the fact that a number of nuclear transport factors have been implicated in cell-size control at mitotic entry [29, 30].

**Figure 3:**
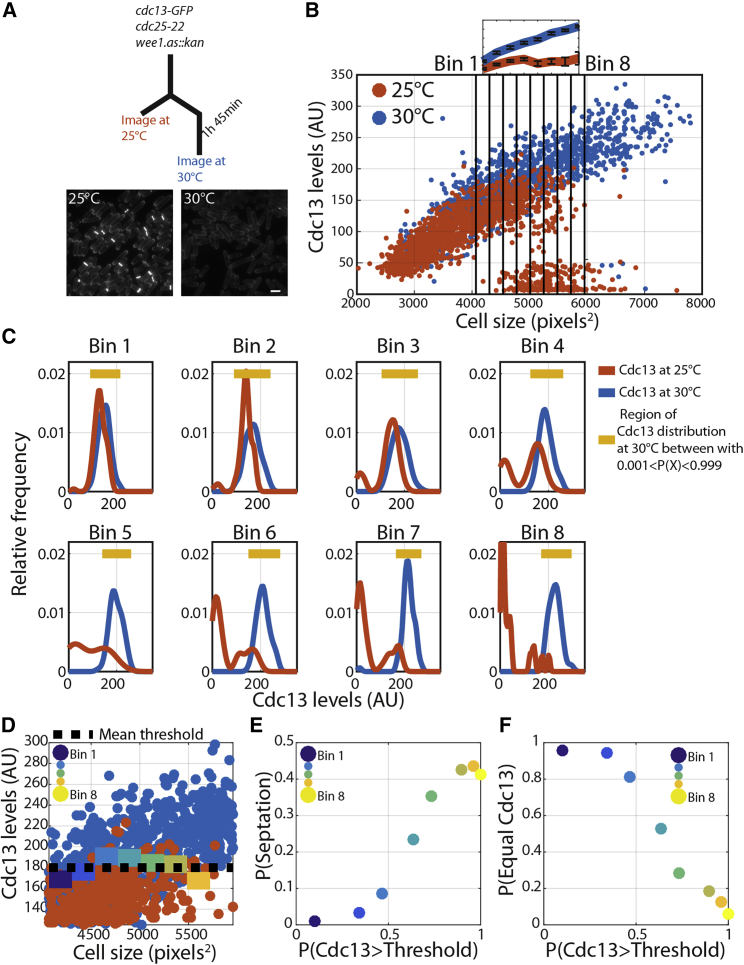
Heterogeneity in Cdc13 Levels Could Explain the Increasing P(Div) with Cell Size (A) Schematic of the experiment. Cells are shifted to 30°C for a short period of time to inhibit division (see example image). The scale bar indicates 10 μm. (B) Scatterplot of Cdc13 levels versus cell size. The subplot above the large plot indicates mean Cdc13 levels within the 8 annotated bins. Error bars indicate the standard deviation of data within the bin. N > 500 cells for each condition. (C) Relative frequency plots of Cdc13-sfGFP levels within annotated size bins in (B), and relative frequency plots are kernel density plots. (D) Scatterplot of Cdc13 fluorescence versus size focused on the region containing the dividing cell. Solid, colored lines represent the calculated within-size-bin Cdc13 threshold required to trigger anaphase (calculated as the mean of the brightest 10 cells within the bin). The dashed black line represents the calculated mean threshold of the intra-size-bin thresholds (180 AU). (E) P(Septation) is calculated as the proportion of cells within the size bin at 25°C that feature a septum. The scatterplot of P(Septation) versus P(Cdc13 > Threshold) is shown, with color indicating the corresponding size bin. (F) P(Equal Cdc13) is calculated as the proportion the 25°C Cdc13-sfGFP population with intensities falling within the yellow bar in (C)—purple line. P(Cdc13 > Threshold) is calculated as the proportion of cells at 30°C with Cdc13 levels >180 AU. The scatterplot of P(Equal Cdc13) versus P(Cdc13 > Threshold) is shown, with color indicating the corresponding size bin. See also Table S1 and Data S1.

Cdc13-sfGFP are variable even in cells of similar size, which is likely to be due to general stochastic processes involved in gene expression [31]. Additionally, it is not possible to determine the level of Cdc13 in mitosis, because Cdc13 triggers its own degradation through activation of the APC/C [32, 33]. To overcome this difficulty and test whether the molecular heterogeneity in Cdc13 levels predicts the decision to enter mitosis, we made use of a Cdc13-sfGFP strain with a *cdc25-22* mutation in the background. Shifting this strain to 30°C for just a short period before imaging temporarily prevents cell division, so all cells present in the population are uninucleates born with the same size distribution as the population at 25°C (Figure 3A). At 30°C, Cdc13 continues to accumulate, but at 25°C, Cdc13-sfGFP levels plateau over the region where cells are dividing (Figure 3B). This plateau could indicate that a threshold Cdc13 level is required for cell division. If cells that stochastically contain suprathreshold levels of cyclin within a size bin are the cells that subsequently enter mitosis and divide, this should be revealed by the degree of overlap between Cdc13-sfGFP distributions at 25°C (division permitted) and 30°C (division inhibited). Cells were binned into 8 size bins across the range of cell sizes that undergo division at 25°C. As cell size increases, there is a decrease in the fraction of dividing cells, with Cdc13-sfGFP levels overlapping with the non-dividing Cdc13-sfGFP distribution (Figure 3C). This decrease is due to the mean of the non-dividing Cdc13-sfGFP distribution increasing, while almost none of the cells in the dividing population feature Cdc13-sfGFP levels above a certain level. We calculated a threshold Cdc13 level required for division at 25°C (Figure 3D). Using this threshold, we determined the fraction of non-dividing cells (30°C) that would be expected to have triggered division were they at 25°C. The proportion of suprathreshold cells within a size bin correlated well with the fraction of septated cells at 25°C (Figure 3E). The fraction of cells in the dividing population of cells that have Cdc13 levels that overlap with the non-dividing population (a measure of anaphase entry) is anti-correlated with the proportion of suprathreshold cells at 30°C (Figure 3F). This supports the hypothesis that cells that have suprathreshold levels of Cdc13 at 25°C are the ones that divide.

We conclude that Cdc13 rises noisily in mean concentration level as cells grow and that Cdc13 must hit a threshold concentration required for division to be triggered. Due to the noise in the Cdc13 level, a fraction of small cells may have suprathreshold Cdc13 levels and thus may trigger division. In larger cells, almost all cells have suprathreshold Cdc13 levels, and thus there is a high frequency of division. This molecular heterogeneity in Cdc13 concentration coupled to a Wee1/Cdc25-dependent Cdc13 threshold could form the molecular basis of the P(Div) size model.

**Figure 4:**
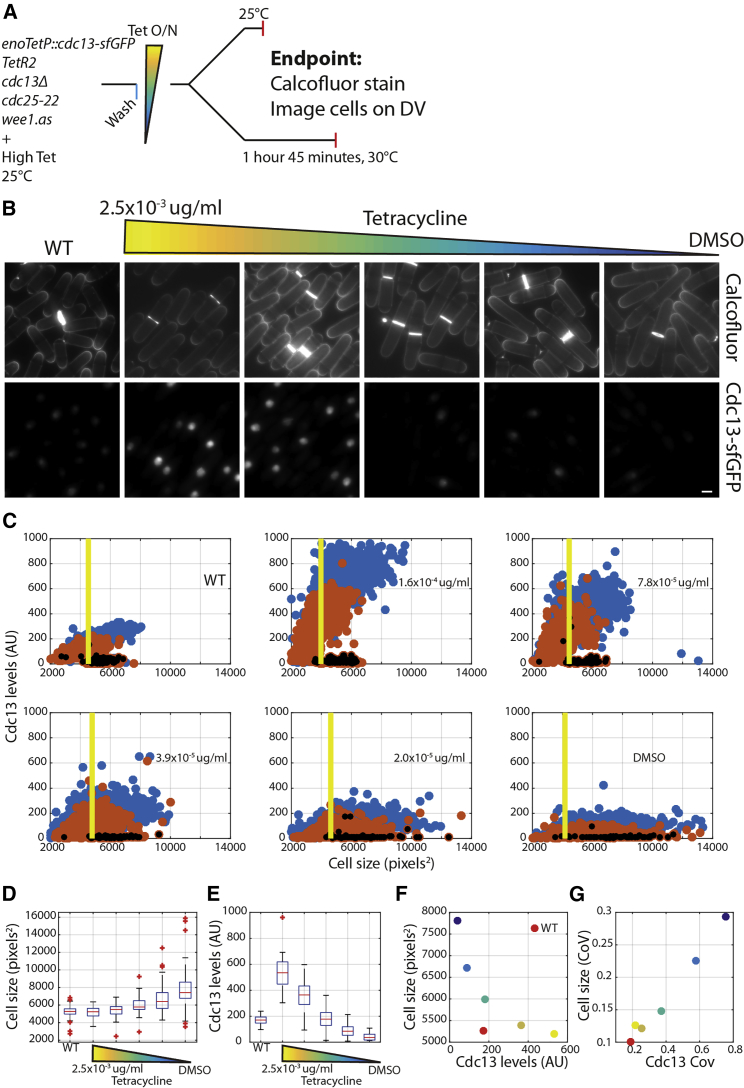
Variability in Cdc13 Levels Is Rate Limiting for Cell-Size Fidelity at G2/M (A) Schematic of the experiment. Cells are grown in EMM+L to steady state at 25°C and are subsequently washed and grown in 2.5 × 10^−3^ μg/mL tet overnight before imaging. (B) Example stills from imaged cells. The scale bar indicates 10 μm. (C) Scatterplots of Cdc13-sfGFP levels versus cell size in WT and tet-dosed cells. The yellow bar is placed at the 1^st^ decile of cell size at division. N > 500 cells for each condition. Black overlaid data points indicate septation. Red data points, 25°C; blue data points, 30°C. (D) Boxplot of cell size at division in annotated strains and tet concentrations. (E) Boxplot Cdc13-sfGFP levels in 30°C condition at size corresponding to yellow line in (B) +/−100 pixels^2^. (F) Plot of 1^st^ decile of cell size at division versus mean Cdc13-sfGFP levels from distribution in (D) and (E). Colors indicate the tet concentration (and the red point indicates the WT cell). (G) Plot of CoV of size at division versus CoV of Cdc13-sfGFP levels from distribution in (D) and (E). Colors indicate the tet concentration (and the red point indicates the WT cell). See also Figures S3 and S4, Table S1, and Data S1.

Cdc13 is one of few cell-cycle proteins that has been shown to be haploinsufficient, with a Cdc13 heterozygous deletion diploid dividing ∼17% longer [30] than WT. The dosing of Cdc13 level against cell size has not been performed, and to investigate this, we engineered a strain expressing Cdc13-sfGFP controlled by a new inducible promoter modulated using anhydrotetracycline (tet) (Figure S3), which features high maximum expression and a linear dose response over an extended range of expression. Imaging was performed at 25°C, and after a brief 30°C shift up in a *cdc25-22* background as above, to reveal cyclin levels in cells that would otherwise be dividing (Figure 4A).

Lower tet concentrations result in lower Cdc13-sfGFP levels and concomitant increases in cell size (Figures 4B–4E). Cdc13-sfGFP levels still scale with cell size, indicating that Cdc13 size scaling could largely be due to its need to reaccumulate after degradation at anaphase rather than a specific size-dependent promoter level regulation of its expression (as has been suggested for Cdc25 [6]) (Figure 4C). Such a reaccumulation model would produce reasonably linear size-correlated Cdc13 expression in some parameter regimes, largely due to a time-dependent correlation between size and Cdc13 concentration reaccumulation to a steady state level (Figure S4; STAR Methods). Cdc13 overexpression above WT levels does not make cells smaller, implying that there is some other rate-limiting feature for the entry of cells into mitosis (Figure 4F). This could be due to Cdc2, because it has been shown previously that co-overexpression of Cdc13 and Cdc2 results in small cells, while overexpression of each alone did not [34]. In support of this hypothesis, overexpression of the Cdc13-Cdc2 fusion protein results in reduced cell size [8, 35].

Cdc13 expression levels below WT levels do result in a larger cell size (Figures 4D and 4F). To investigate whether the CoV of Cdc13 expression correlates with the CoV of cell size at division, we measured the Cdc13-sfGFP levels of cells within a size bin set at the lowest decile of dividing cells ±100 pixels^2^, shown by the yellow line in Figure 4C. The CoV of Cdc13 levels and cell size at division scale linearly (Figure 4G), indicating that noise in Cdc13 expression is important for the fidelity of cell size at division. The threshold level of Cdc13 required for division appears to decrease in larger cells. This could potentially be mediated by the size-dependent accumulation of Cdc25 [6, 7] lowering the cyclin threshold required for division in larger cells.

These data provide support for a probabilistic size-control model, while the *in silico* simulations show that increased division probability with cell size generates cell-size homeostasis. We also show that CDK tyrosine phosphorylation can control the shape of the P(Div) curve and that Cdc13 levels scale with cell size—potentially providing molecular correlates of P(Div) and cell size, respectively. Cdc13 expression is noisy, with Cdc13 levels in cells of the same size being variable. The fraction of cells with Cdc13 levels greater than a threshold correlates with the probability of division in cells of that size, potentially forming the probabilistic component of the P(Div) model. As cell size increases, so does the fraction of cells with Cdc13 levels above the threshold required for division. When Cdc13 expression level is reduced, average cell size at division increases, and variable Cdc13 expression with a similar mean level to WT also results in elongated cells. The CoV of Cdc13 expression correlates well with the CoV of cell size at division, indicating that Cdc13 expression variability may contribute to cell-size fidelity. The increased CoV is driven by the lower level of promoter induction rather than by increased variability in cell division size (Figure S3E). Given the conservation of these cell-cycle regulators, this model is likely to be relevant for other eukaryotic cells.

## STAR★Methods

### Key Resources Table

**Table:** 

REAGENT or RESOURCE	SOURCE	IDENTIFIER
**Chemicals, Peptides, and Recombinant Proteins**		

Anhydrotetracycline hydrochloride	Sigma Aldrich	Product# 37919
3-BrB-PP1	Abcam	ab143756
Fluorescent Brightener 28 (Calcofluor)	Sigma Aldrich	F3543
SwaI	New England BioLabs	R0604S
Q5 Site-Directed Mutagenesis Kit	New England BioLabs	E0554S
Gibson Assembly	New England BioLabs	E5510S
gBlocks gene fragments	IDT DNA	Custom product

**Critical Commercial Assays**		

Imaging flow cytometer: Imagestream X Mark II	Amnis	Cat# 100220

**Experimental Models: Organisms/Strains**		

*S. pombe: h- 972*	Nurse lab collection	PN1
*S. pombe: h? wee1.as::kanMX6*	[25]	IH10938
*S. pombe: h+ cdc13-sfGFP*	[26]	JP302
*S. pombe: h? cdc13-sfGFP cdc25-22*	This manuscript	JP318
*S. pombe: h? cdc13-sfGFP wee1-50*	This manuscript	JP319
*S. pombe: h- cdc2-sfGFP::hphMX6*	This manuscript	JP541
*S. pombe: h? cdc2-sfGFP::hphMX6 wee1-50*	This manuscript	JP561
*S. pombe: h? cdc2-sfGFP::hphMX6 cdc25-22*	This manuscript	JP562
*S. pombe: h? cdc13-sfGFP wee1.as::kanMX6 cdc25-22*	This manuscript	JP426
*S. pombe: h? TetR leu1::JPp135 (TG)*	This manuscript	JP511
*S. pombe: h? TetR leu1::JPp162 (EG)*	This manuscript	JP512
*S. pombe: h? TetR leu1::JPp163(EV)*	This manuscript	JP513
*S. pombe: h? TetR1 (JPp138) leu1::JPp135 (TG)*	This manuscript	JP514
*S. pombe: h? TetR1 (JPp138) leu1:: JPp162 (EG)*	This manuscript	JP515
*S. pombe: h? TetR1 (JPp138) leu1::JPp163 (EV)*	This manuscript	JP516
*S. pombe: h? TetR2 (JPp137) leu1::JPp135 (TG)*	This manuscript	JP517
*S. pombe: h? TetR2 (JPp137) leu1::JPp162 (EG)*	This manuscript	JP518
*S. pombe: h? TetR2 (JPp137) leu1::JPp163 (EV)*	This manuscript	JP519
*S. pombe: h? leu1::enoTetP:cdc13-sfGFP:adh1T (JPp91) cdc13Δ::natMX6 cdc25-22 TetR2 (JPp137) wee1.as::kanMX6*	This manuscript	JP536

**Recombinant DNA**		

Plasmid: Empty vector, hphMX6 (minimal Leu integration, EV)	This manuscript	JPp163
Plasmid: CMVP:TetR-tup11Δ70 (TetR)	[36], Addgene	Cat# 41027
Plasmid: CMVP_TetOx2:TetR-tup11Δ70 (TetR2)	This manuscript	JPp137
Plasmid: CMVP_TetOx1:TetR-tup11Δ70 (TetR1)	This manuscript	JPp138
Plasmid: enotTetP:cdc13-sfGFP:adh1T	This manuscript	JPp91
Plasmid: enoTetP:sfGFP:adh1T(ET)	This manuscript	JPp135
Plasmid: eno101P:sfGFP:adh1T (TG)	This manuscript	JPp162

**Software and Algorithms**		

MATLAB	MathWorks	R2015b
Adder model simulation script: cellAdderTotDivSim	This manuscript	https://github.com/jamesop/CBiol_Patterson2019
Timer model simulation script: cellTimerTotDivSim	This manuscript	https://github.com/jamesop/CBiol_Patterson2019
Sizer model simulation script: cellSizerTotDivSim	This manuscript	https://github.com/jamesop/CBiol_Patterson2019
P(Div) model simulation script: cellPlatTotDivSim	This manuscript	https://github.com/jamesop/CBiol_Patterson2019
Cdc13 accumulation model simulation script: cellCdc13TotDivSim	This manuscript	https://github.com/jamesop/CBiol_Patterson2019

### Contact for Reagent and Resource Sharing

Further information and requests for reagents may be directed to and will be fulfilled by the Lead Contact, James O. Patterson (james.patterson@crick.ac.uk).

### Experimental Model and Subject Details

#### *S. pombe* genetics and cell culture

*S. pombe* media and standard methods are as previously described [37]. After nitrogen and glucose addition, EMM was filter sterilized. This process allows for the generation of clear un-caramelised media. Nutritional supplements for auxotrophic yeast (L-Leucine) was added to a concentration of 0.15 mg/mL. Temperature-sensitive mutant strains were grown at temperatures as specified in the text. To modulate inducible promoters, anhydrotetracycline (Sigma) in DMSO at specified concentrations was used from 1000x stock solutions. To alter Wee1.as activity, 3-BrB-PP1 diluted in methanol was used at a final concentration of 30 μM from a 1000x stock (Abcam). To stain for septa, calcofluor (Fluorescent Brightener 28 (Sigma)) was made up in water at 1 g/L and used as 500x stock.

#### Integration vector construction

A new *leu1* targeting integration plasmid was generated through subcloning via Gibson Assembly (NEB) of a DNA sequence complementary to the *leu1* promoter and terminator with a SwaI restriction enzyme recognition site inserted (generated by commercial DNA synthesis (IDT)) into a pFA6A plasmid with hygromycin resistance [38], generating JPp163. The construct (and dependent constructs) were linearized by digestion with SwaI (NEB) and transformed. Positive colonies can be selected for by Hygromycin resistance, and correctly integrated constructs can be screened for by leucine auxotrophy due to deletion of the *Leu1* ORF. The *Cdc13-sfGFP* ORF was cloned from Jp302 by PCR of the strain and inserted into JPp163 in tandem with the Adh1T and EnoTet promoter by Gibson Assembly (NEB), generating JPp91. Q5 site directed mutagenesis (NEB) was used to delete the components of the Cdc13 ORF from JPp91 to yield JPp135. Finally, JPp162 was generated by subcloning a commercially synthesized (IDT) eno101 promoter fragment [39] into JPp135 by Gibson Assembly (NEB), replacing the enoTet promoter in the process.

#### Tet inducible promoter construction and use

To create a new promoter system for *S. pombe*, a previously defined minimal, strong and small promoter from the eno101 gene was re-engineered [39]. To engineer the capacity for regulation by tet, 3 tet operons were added in the region of the promoter’s TATA box (the enoTet promoter) (Figure S3A). These promoter constructs were generated by commercial DNA synthesis (IDT). The resulting promoter has a strength approximately 34% of the WT promoter (Figure S3B), indicating that it likely has an expression level ∼50% of the nmt1 promoter when fully induced [39].

To express the tet repressor in *S. pombe* a previously published constitutive tetR expression plasmid was used [36]. When co-engineered into a strain expressing GFP from the enoTet promoter, in the absence of tet, expression of GFP is decreased. To define the dose response function of the promoter, a range of tet concentrations were added and cells were grown overnight, followed by GFP measurement on live cells via FACS analysis (Figure S3C).

To engineer a linear dose response function instead of the non-linear one observed with the WT (cytomegalovirus) CMV promoter driven TetR the CMV promoter was re-engineered to contain either one (TetR1) or two (TetR2) tet operons surrounding the TATA box by Q5 site directed mutagenesis (NEB) (Figure S3A). This promoter negative autoregulation strategy has been shown to linearize switch like inducible promoter dose responses in *S. cerevisiae* and human cells [40, 41]. The dose response function was measured and fit to a Hill function, with a Hill coefficient of 10.0 in TetR, to 8.7 in TetR1 and 7.1 in TetR2. This linearization came at the cost of increased background GFP expression, with fold-change expression in TetR of 64, TetR1 of 31, and TetR2 of 17 (Figure S3C).

### Method Details

#### Imaging flow cytometry

Imaging flow cytometry allows for the acquisition of thousands of single cell brightfield and fluorescent images in a short period of time. Imaging flow cytometry was performed using an Imagestream Mark X two-camera system (Amnis), using the 60x objective. Cells were concentrated by centrifugation (5000 rpm/30 s) and resuspended in ∼25 μL of media before sonication in a sonicating water bath. Imaging was restricted to < 10 min to avoid any phenotypic perturbations within the system. Cells were gated pre-acquisition using:

1. Gradient RMS > 65 (a measure of cell focus).
2. Area/Aspect ratios consistent with single cells.

To avoid any autofocus based drift within an experiment, cell were imaged at fixed, empirically determined focal points, designed to maximize the number of cells with gradient RMS > 65. Data was analyzed using custom MATLAB scripts.

#### Microscopic imaging

All imaging was performed using a Deltavision Elite (Applied Precision) microscope – an Olympus IX71 wide-field inverted fluorescence microscope with a PLAN APO 60x oil, 1.42 NA objective and a Photometrics CoolSNAP HQ2 camera. To maintain specified temperatures during imaging, an IMSOL imcubator Environment control system and an objective heater was used. SoftWoRx was used to set up experiments. 5 z stacks were acquired, with 1 μm spacing. Image analysis was performed using custom MATLAB scripts.

Slides for live cell steady state imaging were prepared by pelleting 1 mL of > 6x10^6^ cells/mL of culture. The pellet was then resuspended in media (or media+calcofluor) in ∼3.5 μL before 1.5 μL of this suspension was applied to a glass slide and covered with a coverslip. Fields of view were avoided that contained dead cells due to compression by the coverslip and were otherwise sampled at random. 10-20 FOV were acquired per slide, for < 15 min to avoid imaging any perturbations resulting from slide-based acquisition. Imaging was optimized for signal intensity, as each FOV was only used once and thus photobleaching and photo-toxicity were not of concern.

#### *In silico* modeling

Modeling was performed in MATLAB, through simple numerical simulation. Cell growth was modeled as exponential, and each cycle of the model corresponded to 1 min real time. Cells were grown on each cycle by adding length to them, with the amount added predicted by an exponential model of cell growth. Cells were triggered to divide based on specific rules for whatever model was being tested. We are happy to provide the scripts used on request. Initial cell populations were generated by initialising a group of cells with size varied around a mean. On division, new born cells are generated with a size 1.05^∗^(Division size/2), and minimal asymmetry is generated by adding Gaussian noise of mean 0, standard deviation 1, to one daughter cell and subtracting it from the other daughter cell.

##### For the adder model simulation, the following parameters and rule sets were used

Division rule: Once a cell has grown a specified amount, division is triggered.
Increase in size versus current size/min: 0.006.
Initial number of cells: 20.
Time of simulation: 1000 cycles (minutes).
Threshold size required to be added to trigger division: 6.75 units.
Standard deviation of adder threshold: 1 unit.
Initial size of population sampled from Gaussian with mean of 6.75 and standard deviation of 0.675.

##### For the sizer model simulation, the following parameters and rule sets were used

Division rule: Once a cell has reached a certain size, division is triggered.
Increase in size versus current size/min: 0.006.
Initial number of cells: 20.
Time of simulation: 1000 cycles (minutes).
Threshold size required to trigger division: 14.5 units.
Standard deviation of sizer threshold: 1.2 units.
Initial size of population sampled from Gaussian with mean of 7.25 and standard deviation of 0.725.

##### For the timer model simulation, the following parameters and rule sets were used

Division rule: Once a cell has been growing for a certain threshold time, division is triggered.
Increase in size versus current size/min: 0.006.
Initial number of cells: 20.
Time of simulation: 1000 cycles (minutes).
Threshold time required to trigger division: 100 min.
Standard deviation of sizer threshold: 5 min.
Initial size of population sampled from Gaussian with mean of 7 and standard deviation of 0.7.

##### For the P(Div) model simulation, the following parameters and rule sets were used

Division rule: Each cycle of the simulation, the size dependent probability of division is sampled from the P(Div) curve. Each cell is assigned a random number from 0-1 from a Gaussian distribution. If the random number is below the predicted P(Div), then division is triggered.
Increase in size versus current size/min: 0.0029.
Initial number of cells: 20.
Time of simulation: 1000 cycles (minutes).
P(Div) curve parameters: Minimum probability, 0. Maximum probability, 0.1. EC_50_, 14. Hill coefficient, 14.
Initial size of population sampled from Gaussian with mean of 14 and standard deviation of 1.4.

##### For the Cdc13 accumulation model simulation, the following parameters and rule sets were used

Division rule: Once a cell has accumulated a threshold concentration of Cdc13, division is triggered. At 500 min, an unattainable threshold Cdc13 concentration for division was set to observe Cdc13 accumulation in non-dividing cells.
Increase in size versus current size/min: 0.006.
Initial number of cells: 10.
Time of simulation: 1000 cycles (minutes).
Amount of Cdc13 synthesized per minute: 0.07 units.
Noise in amount of Cdc13 synthesized per minute: 0.05 units.
Amount of Cdc13 degraded per minute: 0.0001 units.
Cdc13 threshold level for division: 6 units.
Noise in Cdc13 threshold level for division: 0.3 units.
Cdc13 level in a cell on birth: Birth size^∗^0.7.
Initial size of population sampled from Gaussian with mean of 7 and standard deviation of 0.7.

#### Experimental design

Figure 1B was generated from data with biological replicates n = 3 and pooled. Subsequent data was based on individual cells with sample size detailed in Table S1 (in all cases > 400) and n = 1. Only cells that featured segmentation errors were excluded from analysis, as judged by manual screening of images. Blinding and randomization were not performed.

### Quantification and Statistical Analysis

Box-and-whisker plots are delimited by 25^th^ percentile, median and 75^th^ percentile. Whisker lengths are either the distance to the furthest point outside of the box, or 1.5x the interquartile range, whichever is lower. If data exists that is greater than 1.5x the interquartile range from the top or bottom of the box, this is shown as a red “+.”

P(Div)/min calculation was performed by normalizing the non-rate P(Div) function by septation time. As septation time does not scale with cell size [15], the number of cells that are septated versus non-septated in a population is related to both the amount of time cells spend septated and the probability of cells of that size septating. Thus, to septation rate can be extracted by dividing the P(Div) curve by the time cells spent septated (∼30 min), thus yielding a P(Div) rate curve with units min^-1^.

Statistical testing was not performed. Exact sample sizes for each experiment can be found in Table S1.

### Data and Software availability

Data used in Figure 1B, Figures 2D and 2E, Figure 3, and Figure 4 are available in Data S1. MATLAB scripts used for modeling and simulation of cell size control models described are available at https://github.com/jamesop/CBiol_Patterson2019.

## Supplemental Information

Document S1. Figures S1–S4 and Table S1

Data S1. Experimental Data, Related to Figures 1B, 2D, 2E, 3, and 4

Document S2. Article plus Supplemental Information
