# Supplementary material for: Noisy Cell-Size-Correlated Expression of Cyclin B Drives Probabilistic Cell-Size Homeostasis in Fission Yeast
Source: Curr Biol. 2019 Apr 22;29(8):1379–1386.e4. doi: 10.1016/j.cub.2019.03.011 (PMC6488275; doi:10.1016/j.cub.2019.03.011)
Supplement: Document S1. Figures S1–S4 and Table S1 [file mmc1.pdf]

Current Biology, Volume *29*

Supplemental Information

Noisy Cell-Size-Correlated Expression of Cyclin B Drives Probabilistic Cell-Size Homeostasis in Fission Yeast

James O. Patterson, Paul Rees, and Paul Nurse

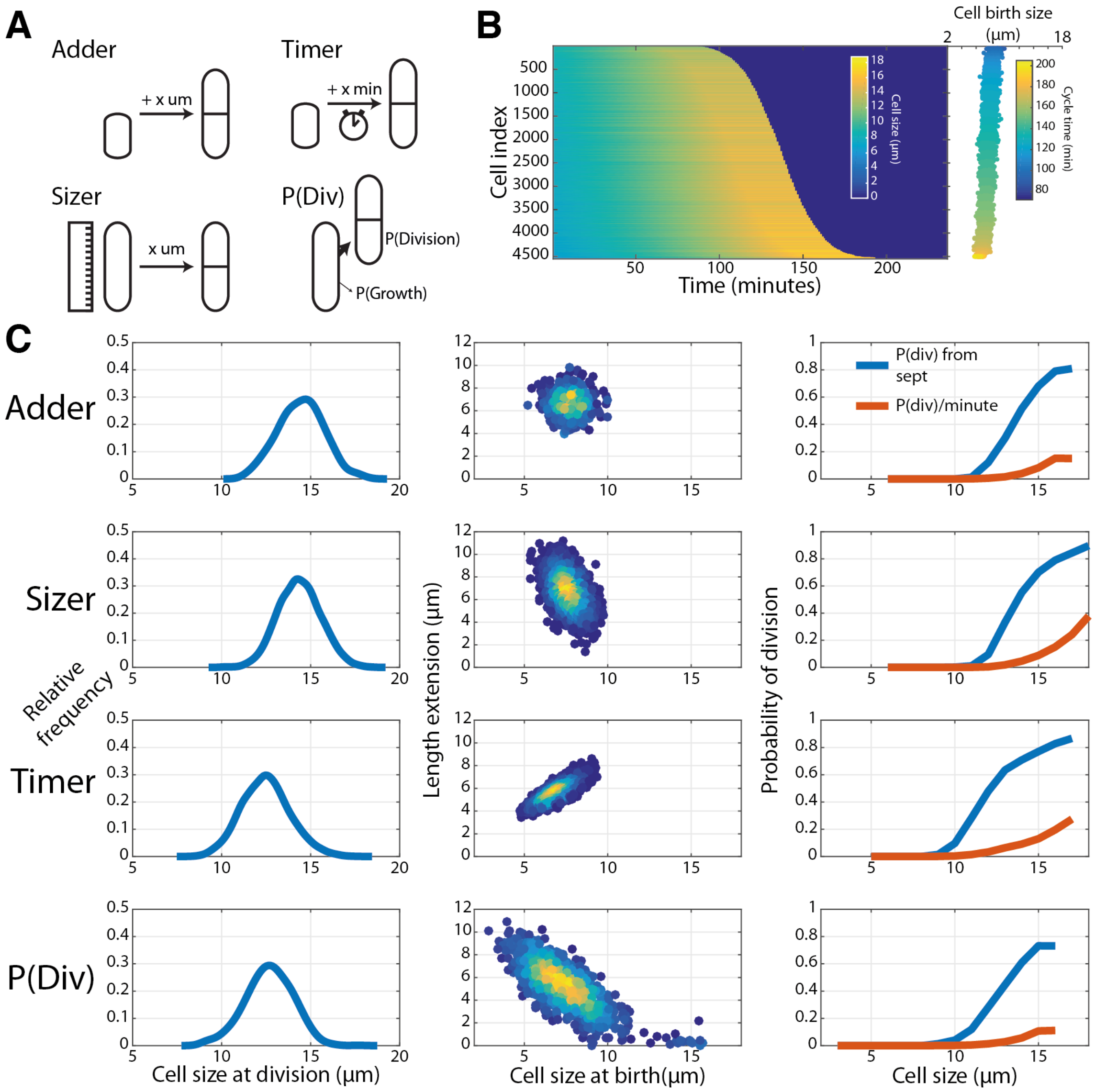

Figure S1. Comparison of simulated cell size control mechanisms, Related to Figure 1

A Schematics of various commonly proposed size control models (adder, timer and sizer) in addition to our new model, P(Div).

B Example heatmap of *in silico* generated cell length traces, with a P(Div) model used for triggering division. Scatter plot to the right of heatmap indicates cell birth size. Cell index is ordered by cycle time.

C Cell size control features extracted from *in silico* data. All simulations are initiated with 20 cells roughly at the mean birth size, and run for 1000 minutes. All cells grow

according to an exponential function which results in size doubling within ~120 minutes. All simulations result in >1000 individual full cell cycles.

Adder model: size added is sampled randomly from a distribution with mean 6.75 and standard deviation of 1.

Deterministic sizer model: size at division for any cell is sampled from distribution with mean 14.5 and standard deviation of 1.2

Timer model: cell cycle time is sampled randomly from a distribution with mean 100 and standard deviation of 5.

P(Div) size model: Probability of cell division at a certain cell size is sampled from a Hill curve with maximum probability 0.1, EC50 of 14 and Hill coefficient of 14.

Fantes plots are coloured by local density of points (dark blue – low, yellow – high).

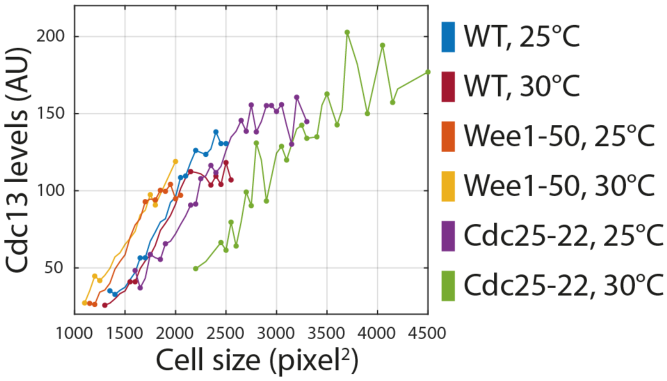

Figure S2. Average Cdc13 levels plateau at different heights in different cell size mutants, Related to Figure 2

Lines correspond to mean Cdc13-sfGFP levels of size binned data with window size 100 pixels^2. Scatter data is shown in Figure 2.

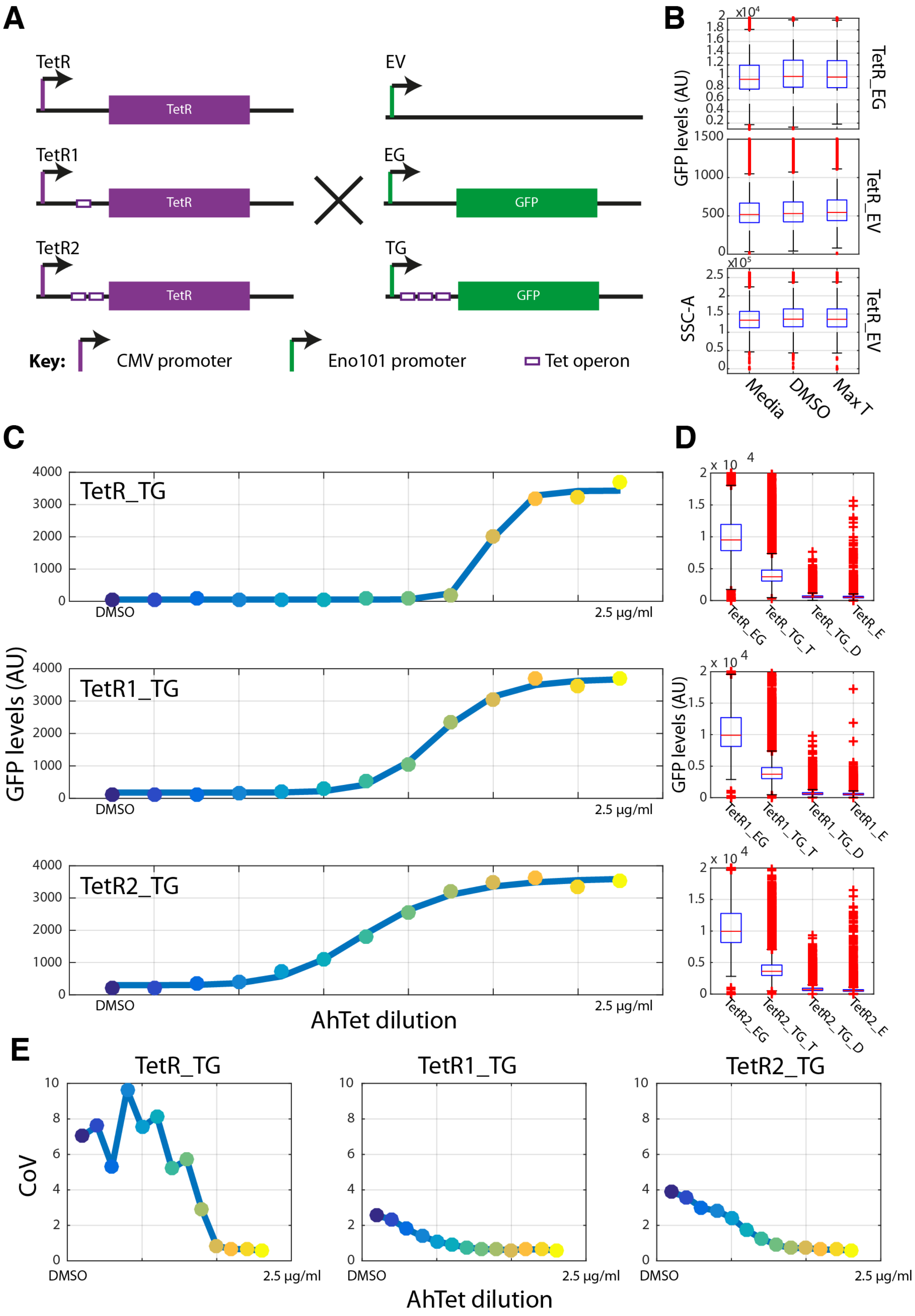

A
TetR
TetR1
TetR2
EV
EG
TG
GFP
Key:
CMV promoter
Eno101 promoter
Tet operon
B
GFP levels (AU)
SSC-A
TetR_EG
TetR_EV
Media
DMSO
Max T
C
TetR_TG
TetR1_TG
TetR2_TG
GFP levels (AU)
DMSO
2.5 µg/ml
AhTet dilution
D
TetR_EG
TetR_TG_T
TetR_TG_D
TetR_E
TetR1_EG
TetR1_TG_T
TetR1_TG_D
TetR1_E
TetR2_EG
TetR2_TG_T
TetR2_TG_D
TetR2_E
E
CoV
AhTet dilution

Figure S3. A new synthetic tet regulatable expression system for *S. pombe*, Related to Figure 4, Related to STAR Methods

A Schematic of tet repressor and tet promoter constructs. The base TetR system has been published previously [S1], however TetR1 and TetR2 are presented here for the first time. EG has been shown previously [S2], and TG was developed here.

B Boxplots of GFP levels and cell size in control strains demonstrating that neither DMSO or tet have any effect on cell size or background GFP fluorescence.

C Plots of GFP levels vs tet concentration in annotated strains. Measured using FACS analysis. Solid lines are Hill function fit to mean data. Data points are mean data. Hill coefficient are 10.0 for TetR, 8.7 for TetR1 and 7.1 for TetR2. Fold-change expression with TetR was 64, TetR1 was 31, and TetR2 was 17. Colours indicate tet dilutions. N~50,000 cells per condition.

D Boxplots of GFP expression levels in annotated strains.

E Plots of the Coefficient of Variation (CoV) vs tet concentration in annotated strains. Background subtraction was performed before CoV calculation.

See also Table S1.

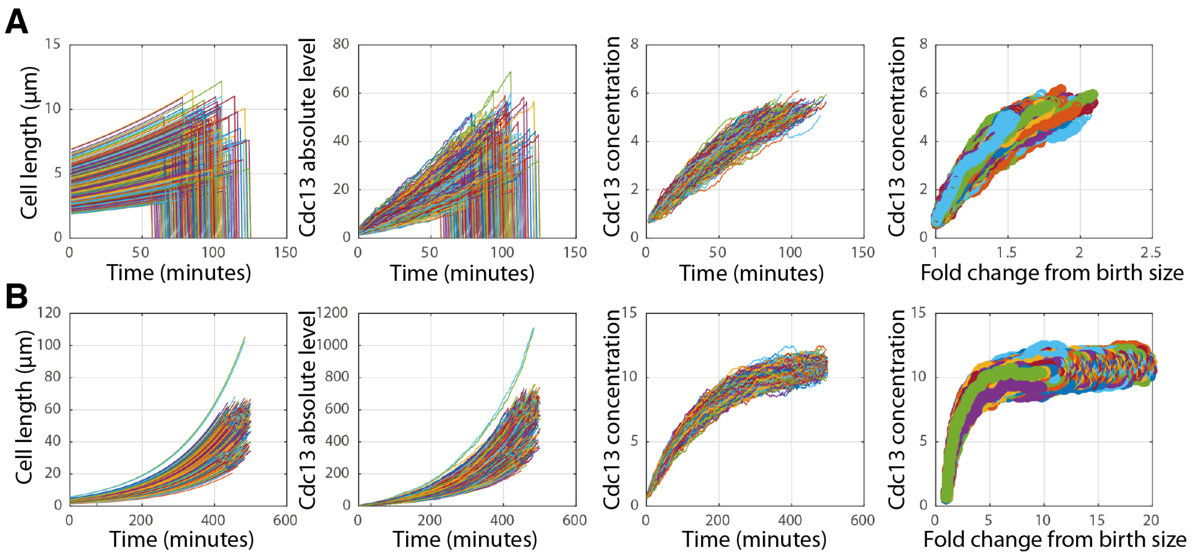

Figure S4. Cdc13 reaccumulation to a steady state concentration after cell birth allows for scaling with cell length, Related to Figure 4

A Cell growth was modelled to be exponential as previously done. However, in this model, Cdc13 molecules were produced at a size dependent rate (as all proteins must be to maintain them at a constant concentration during cell growth [S3]). Cell division was set to occur at a set Cdc13 *concentration* threshold (with added noise). On new cell birth, Cdc13 levels were initialised in proportion to cell birth size (note that larger cell mutants have more Cdc13 initially in Figure S2). Note that the scatter plot of cell size increase vs. Cdc13 concentration demonstrates an approximately linear relationship.

B Cell growth and Cdc13 accumulation were modelled with the same parameters as in **A** however division was not permitted. Here, Cdc13 concentration increases to a size independent constant level, indicating it has reaccumulated to its set point.

Figure	Imaging technique	Strain	Number of cells	Treatment
**Figure 1B:**	Imagestream	PN1	275087	25°C, Calcofluor staining
**Figure 2C:**	Imagestream	wee1.as::kanMX6	>1000/Timepoint	25°C, Calcofluor staining and 3BRB-PP1 addition (30 micromolar)
**Figure 2D:**	Widefield microscopy	JP302	1136	25°C, Calcofluor staining
	Widefield microscopy	JP318	1241	25°C, Calcofluor staining
	Widefield microscopy	JP319	1382	25°C, Calcofluor staining
	Widefield microscopy	JP302	1429	30°C temperature shift for 6 hours, Calcofluor staining
	Widefield microscopy	JP318	1128	30°C temperature shift for 6 hours, Calcofluor staining
	Widefield microscopy	JP319	555	30°C temperature shift for 6 hours, Calcofluor staining
**Figure 2E:**	Widefield microscopy	JP541	1833	25°C, Calcofluor staining
	Widefield microscopy	JP561	1651	25°C, Calcofluor staining
	Widefield microscopy	JP562	1745	25°C, Calcofluor staining
	Widefield microscopy	JP541	1964	30°C temperature shift for 6 hours, Calcofluor staining
	Widefield microscopy	JP561	1326	30°C temperature shift for 6 hours, Calcofluor staining
	Widefield microscopy	JP562	428	30°C temperature shift for 6 hours, Calcofluor staining
**Figure 3:**	Widefield microscopy	JP426	1608	25°C, Calcofluor staining
	Widefield microscopy	JP426	1354	30°C for 1 hour 45 minutes, Calcofluor staining
**Figure 4:**	Widefield microscopy	JP426	1608	25°C, Calcofluor staining
	Widefield microscopy	JP536	931	1.6E-4 ug/ml tet, 25°C, Calcofluor staining

	Widefield microscopy	JP536	886	7.8E-5 ug/ml tet, 25°C, Calcofluor staining
	Widefield microscopy	JP536	782	3.9E-5 ug/ml tet, 25°C, Calcofluor staining
	Widefield microscopy	JP536	736	2.0E-5 ug/ml tet, 25°C, Calcofluor staining
	Widefield microscopy	JP536	753	DMSO, 25°C, Calcofluor staining
	Widefield microscopy	JP426	1354	30°C for 1 hour 45 minutes, Calcofluor staining
	Widefield microscopy	JP536	998	1.6E-4 ug/ml tet, 30°C for 1 hour 45 minutes, Calcofluor staining
	Widefield microscopy	JP536	1169	7.8E-5 ug/ml tet, 30°C for 1 hour 45 minutes, Calcofluor staining
	Widefield microscopy	JP536	1391	3.9E-5 ug/ml tet, 30°C for 1 hour 45 minutes, Calcofluor staining
	Widefield microscopy	JP536	1271	2.0E-5 ug/ml tet, 30°C for 1 hour 45 minutes, Calcofluor staining
	Widefield microscopy	JP536	690	DMSO, 30°C for 1 hour 45 minutes, Calcofluor staining
**Figure S3**	FACS BD Fortessa	Various	50,000 cells/condition	25°C, DMSO/Tetracycline/EMM+L treatment

Table S1. Experimental details, Related to Figures 1, 2, 3, 4 and S3

Experimental conditions, cell numbers, and strains used in each experiment.

Supplemental References

S1. Zilio, N., Wehrkamp-Richter, S., and Boddy, M.N. (2012). A new versatile system for rapid control of gene expression in the fission yeast *Schizosaccharomyces pombe*. Yeast *29*, 425–434.

S2. Wang, H., Wang, H., Wang, M., Zhang, L., Wang, R., Mei, Y., and Shao, W. (2014). Identification and refinement of two strong constitutive promoters for gene expression system of Schizosaccharomyces pombe. World J. Microbiol. Biotechnol. *30*, 1809–1817.

S3. Zhurinsky, J., Leonhard, K., Watt, S., Marguerat, S., Bähler, J., and Nurse, P. (2010). A Coordinated Global Control over Cellular Transcription. Curr. Biol. *20*, 2010–2015.
